# The Global Gridded Crop Model Intercomparison phase 1 simulation dataset

**DOI:** 10.1038/s41597-019-0023-8

**Published:** 2019-05-08

**Authors:** Christoph Müller, Joshua Elliott, David Kelly, Almut Arneth, Juraj Balkovic, Philippe Ciais, Delphine Deryng, Christian Folberth, Steven Hoek, Roberto C. Izaurralde, Curtis D. Jones, Nikolay Khabarov, Peter Lawrence, Wenfeng Liu, Stefan Olin, Thomas A. M. Pugh, Ashwan Reddy, Cynthia Rosenzweig, Alex C. Ruane, Gen Sakurai, Erwin Schmid, Rastislav Skalsky, Xuhui Wang, Allard de Wit, Hong Yang

**Affiliations:** 1 Potsdam Institute for Climate Impact Research, Member of the Leibniz Association, 14473 Potsdam, Germany; 2 University of Chicago and ANL Computation Institute, Chicago, IL 60637 USA; 3 Karlsruhe Institute of Technology, IMK-IFU, 82467 Garmisch-Partenkirchen, Germany; 4 Ecosystem Services and Management Program, International Institute for Applied Systems Analysis, 2361 Laxenburg, Austria; 5 Department of Soil Science, Comenius University in Bratislava, 842 15 Bratislava, Slovak Republic; 6 Laboratoire des Sciences du Climat et de l’Environnement, CEA CNRS UVSQ Orme des Merisiers, F-91191 Gif-sur-Yvette, France; 7 Center for Climate Systems Research, Columbia University, New York, NY 10025 USA; 8 Earth Observation and Environmental Informatics, Alterra Wageningen University and Research Centre, 6708PB Wageningen, Netherlands; 9 Department of Geographical Sciences, University of Maryland, College Park, MD 20742 USA; 10 Texas AgriLife Research and Extension, Texas A&M University, Temple, TX 76502 USA; 11 Earth System Laboratory, National Center for Atmospheric Research, Boulder, CO 80307 USA; 12 Eawag, Swiss Federal Institute of Aquatic Science and Technology, CH-8600 Duebendorf, Switzerland; 13 Department of Physical Geography and Ecosystem Science, Lund University, 223 62 Lund, Sweden; 14 School of Geography, Earth & Environmental Science, University of Birmingham, Edgbaston, Birmingham, B15 2TT United Kingdom; 15 Birmingham Institute of Forest Research, University of Birmingham, Edgbaston, Birmingham, B15 2TT United Kingdom; 16 National Aeronautics and Space Administration Goddard Institute for Space Studies, New York, NY 10025 USA; 17 Institute for Agro-Environmental Sciences, National Agriculture and Research Organization, Tsukuba, 305-8604 Japan; 18 Institute for Sustainable Economic Development, University of Natural Resources and Life Sciences, 1180 Vienna, Austria; 19 Soil Science and Conservation Research Institute, National Agricultural and Food Centre, 82109 Bratislava, Slovak Republic; 20 Sino-French Institute of Earth System Sciences, Peking University, 100871 Beijing, China; 21 Department of Environmental Sciences, MGU, University of Basel, CH-4003 Basel, Switzerland

**Keywords:** Plant sciences, Computational models, Environmental sciences, Agroecology

## Abstract

The Global Gridded Crop Model Intercomparison (GGCMI) phase 1 dataset of the Agricultural Model Intercomparison and Improvement Project (AgMIP) provides an unprecedentedly large dataset of crop model simulations covering the global ice-free land surface. The dataset consists of annual data fields at a spatial resolution of 0.5 arc-degree longitude and latitude. Fourteen crop modeling groups provided output for up to 11 historical input datasets spanning 1901 to 2012, and for up to three different management harmonization levels. Each group submitted data for up to 15 different crops and for up to 14 output variables. All simulations were conducted for purely rainfed and near-perfectly irrigated conditions on all land areas irrespective of whether the crop or irrigation system is currently used there. With the publication of the GGCMI phase 1 dataset we aim to promote further analyses and understanding of crop model performance, potential relationships between productivity and environmental impacts, and insights on how to further improve global gridded crop model frameworks. We describe dataset characteristics and individual model setup narratives.

## Abstract (summary)

**Table:** 

Design Type(s)	modeling and simulation objective • data integration objective • software variation design • time series design
Measurement Type(s)	cultivated environment
Technology Type(s)	computational modeling technique
Factor Type(s)	crop • atmospheric weather • atmospheric wind speed • atmospheric water vapour • atmospheric carbon dioxide • agricultural environmental material • agricultural feature
Sample Characteristic(s)	land

Machine-accessible metadata file describing the reported data (ISA-Tab format)

## Background & Summary

Croplands cover about 11% of the total land area and are responsible for most of the 60% of anthropogenic nitrous oxide (N_2_O) emissions that are attributed to agriculture and 11% of the anthropogenic methane emissions from rice production^1^, summing up to 4.5% of the total anthropogenic greenhouse gas emissions^2^. Croplands are subject to climate change impacts^3^, land-use change^4,5^, climate mitigation strategies^6,7^, interact directly with the climate system^8^, consume large quantities of human freshwater withdrawals^9^ and are connected to various sustainable development goals^10^. Understanding and quantifying cropland dynamics is thus an integral research question for Earth System Science.

Future agricultural production faces several challenges that need to be understood in scope and implications: (1) growing and increasingly wealthier populations are projected to demand more and different compositions of food commodities^11,12^, (2) climate change impacts^3,13^ will require adaptation^14–18^, and (3) the environmental impact of agricultural production needs to be reduced, including pollution^19,20^, water consumption^9^, land consumption^21,22^ and greenhouse gas emissions^23–25^. The potential to address these challenges is often explored with computer simulation models of agricultural productivity or outputs of such agricultural productivity models in combination with other models, e.g. economic models of the agricultural sector^5^ or hydrology models^26^.

AgMIP (Agricultural Model Intercomparison and Improvement Project, see Table 1 for a complete list of abbreviations) was initiated to help improving agricultural modeling capacities across scales and aspects (soils, different crops, economics etc.) by intercomparing models in simulation experiments using common protocols^27^. The general idea of AgMIP is that different modeling groups around the world can participate, contributing data to the ensemble dataset. Protocols are developed to clearly describe important aspects in the configuration of the modeling experiments and all prescribed inputs are supplied to interested modelers. AgMIP analyses typically start out by describing the range of model results and thus quantifying the uncertainty embedded in the choice of the crop model used and its parameterization. This source of uncertainty has previously not gained much attention. Different groups using the same model are explicitly invited to participate, which allows for analyzing how important modelers’ choices are^28^ beyond model configurations as specified in the protocol or where protocol instructions were not implemented correctly.

**Table 1:** Acronyms used in article.

Abbreviation	Explanation
AgCFSR	Agriculture Climate Forecast System Reanalysis
AgMERRA	Agriculture Modern-Era Retrospective Analysis for Research and Applications
AgMIP	Agricultural Model Intercomparison and Improvement Project
AgroIBIS	agricultural version of the Integrated Biosphere Simulator
APAR	absorbed photosynthetically active radiation
CERES	Crop Environment Resource Synthesis
CESM1	Community Earth System Model
CGMS	European Crop Growth Monitoring System
CLM	Community Land Model
CLM4CN model	Community Land Model version 4.0 carbon nitrogen model
CROPGRO	physiologically-based crop model
CRU	Climate Research Unit
EPIC	Environmental Policy Integrated Climate model
EPIC-BOKU	EPIC model run by the University of Natural Resources and Life Sciences Vienna (BOKU)
EPIC-IIASA	EPIC model run by the International Institute for Applied Systems Analysis
EPIC-TAMU	EPIC model run by the Texas A&M University
FertiSTAT	database for statistics on fertilizer use by crop
FPAR	Fraction of Photosynthetically Active Radiation absorbed by the canopy
GEPIC	Geographic Information Ssystem-based EPIC model
GGCMI	Global Gridded Crop Model Intercomparison
GGCMs	Global Gridded Crop Models
GPCC	Global Precipitation Climatology Centre
GSWP3	Global Soil Wetness Project Phase 3
ISIMIP	Inter-Sectoral Impact Model Intercomparison Project (www.isimip.org)
LAI	Leaf-Area Index
LPJ-GUESS	Lund Potsdam Jena General Ecosystem Simulator
LPJmL	Lund Potsdam Jena managed Land
ORCHIDEE-crop	Organising Carbon and Hydrology In Dynamic Ecosystems crop model
P1 crops	Priority one crops: maize, wheat, rice, soybean
P2 crops	Priority two crops: additional crops
pAPSIM	parallel version of the Agricultural Production Systems sIMulator
pDSSAT	parallel version of the Decision Support System for Agrotechnology Transfer
PEGASUS	Predicting Ecosystem Goods And Services Using Scenarios Model
PEPIC	Python based EPIC model
PGFv2 dataset	Princeton GF version two that spans 1901 - 2012
PHUs	Potential Heat Units
PRYSBI2	Process-based Regional-scale crop Yield Simulator with Bayesian Inference version 2
pSIMS platform	parallel System for Integrating impact Models and Sectors
SWAT	Soil Water Assessment Tool
WATCH	Water and Global Change project
WFDEI	WATCH Forcing Data methodology applied to ERA‐Interim data
WFDEI.CRU	WFDEI; precipitation bias-corrected against CRU
WFDEI.GPCC	WFDEI; precipitation bias-corrected against GPCC
WOFOST	WOrld FOod STudies crop simulation model

Note that some acronyms are only used in specific tables and are only explained there.

Data of the GGCMs provided by AgMIP^27^ in the framework of ISIMIP^29^ have been used to assess impacts of climate change^3,30^, study sources of uncertainties^6,31^ and have been used in combination with other data for cross-sectoral impact analyses^26,32^. The term “cross-sectoral” in the ISIMIP context refers to analyses using data from different impact categories, referred to as “sectors”. The data were also used for economic assessments of climate change impacts on agricultural production systems^5,33–36^. However, this first global-scale simulation ensemble of AgMIP that was conducted as part of the ISIMIP project^3^ revealed a broad range of GGCM results under different climate change scenarios and in response patterns^6,31^. This high level of uncertainty motivated the following GGCMI phase 1 to assess model performance and to identify general fields of model improvement. The protocol for GGCMI phase 1^37^ thus designed a comprehensive modeling exercise aiming to understand GGCM skill in reproducing observed historic crop yield patterns in space and time. Besides inviting a broad group of modeling teams and models, the GGCMI phase 1 protocol also adds variants of management harmonization and weather input datasets. Different assumptions on growing seasons across GGCMs had rendered the initial GGCM simulations^3^ as difficult to compare. The broad availability of different historic weather data products, which also differ substantially in parts^37^, motivated the inclusion of different weather data products to address this source of uncertainty. A comprehensive initial model evaluation study based on the GGCMI phase 1 dataset^38^ showed that GGCMs typically have better skill than statistical models to explain observed crop yield variability but also have little explanatory power in regions where yield variability is mostly driven by changes in management or pest outbreaks, rather than weather variability. None of the GGCMs proved to be generally superior to the others but differences in model skill were reported^38^.The output data from this set of simulations are described here.

The GGCMI phase1 dataset provides an unprecedentedly large dataset of crop model simulations covering the global ice-free land surface. While the dataset has already served various analyses on crop yields^38–41^, there are still many aspects unexplored; variables apart from crop yields have hardly been assessed so far, with the exception of actual growing season evapotranspiration by Wartenburger, *et al*.^42^. The multi-dimensionality of the dataset (14 GGCMs, 11 input datasets, 3 harmonization levels, 4 crops, 14 output variables, time, and space) allows for further analyses and can also serve as an input to other models, the quantification of model uncertainties, and crop model emulation^43,44^. With the publication of the GGCMI phase 1 dataset we aim to promote further analyses and understanding of crop model performance, potential relationships between productivity and environmental impacts (e.g. water, nutrients), and eventually insights on how to further improve global gridded crop model frameworks and configurations.

## Methods

The GGCMI phase 1 dataset^37^ consists of the model output of 14 GGCMs (Table 2) for up to 11 weather datasets covering various time frames and up to 3 harmonization levels (*default*, *fullharm*, *harmnon*) for the 4 priority one (P1) crops (maize, wheat, rice, soybean), as well as for any number of additional crops (priority two, P2). Not all models have been able to simulate all P1 crops and a number of models provided several P2 crop simulations (see Table 3). The GGCMI phase 1 dataset has been compiled by 14 different crop modeling groups that have followed the protocol instructions^37^ to achieve as much consistency as possible. It has to be noted, however, that not everything was or could have been harmonized across models. The focus of harmonization was on weather datasets to drive the models and on a few core crop management settings: the growing period and fertilizer inputs. Many other aspects of crop management have not been specified by the protocol^37^. This is in part owing to the complexity of this task, as the models have very different capacities to represent management options and thus require different sets of parameters. As such, we acknowledge the importance of soil parameters for crop model simulations^45^ but were unable to harmonize on these here. However, the lack of harmonization in various management and soil aspects can also be considered an asset of the analysis. For most regions in the world, the management systems are not documented and typically quite diverse so that the diversity of the assumptions made in the ensemble may better reflect this than a single harmonization target. Folberth *et al*.^46^ indeed show that assumptions made by the different EPIC modeling teams affect the models’ performances and sensitivities.

**Table 2:** GGCMs that provided data to the phase 1 dataset and their main characteristics.

Model	Type^a^	CO_2_ effects^b^	Stresses^c^	Calibration^d^	Calibrated parameters	Key literature
CGMS-WOFOST	Site-based	LF, TE	W, T	Site-specific	PHU requirements	^50^
CLM-crop	Ecosystem	LF, TE	W,N,H	Uncalibrated	NA	^128^
EPIC-BOKU	Site-based	RUE, TE	W, T, H, A, N, P, BD, AL	Site-specific (EPIC 0810)	NA	EPIC v0810 -^60,130^
EPIC-IIASA	Site-based	RUE, TE	W, T, H, A, N, P, BD, AL	Site-specific and global	F, HIpot (ric, mai) F (others)	EPIC v0810 -^60,130^
EPIC-TAMU	Site-based	RUE, TE	W, T, H, A, N, P, BD, AL	Site-specific and global	HIpot (maize)	EPIC v1102 –^65^
GEPIC	Site-based	RUE, TE	W, T, A, N, P, BD, AL	Site-specific (EPIC 0810)	F HIpot (for maize and rice)	EPIC v0810 -^60,66,78,130^
LPJ-GUESS	Ecosystem	LF, SC	W, T	Uncalibrated	NA	^83, 221^
LPJmL	Ecosystem	LF, SC	W, T	National	LAImax HI αa	^77, 84^
ORCHIDEE-crop	Ecosystem	LF, SC	W,T,N	Uncalibrated	—	^94^
pAPSIM	Site-based	RUE	W, T, H, A, N	Site-specific (APSIM)	NA	APSIM v7.5 -^98,99^
pDSSAT	Site-based	RUE (for wheat, rice, maize) and LF (for soybeans)	W, T, H, A, N	Site-specific (DSSAT)	NA	pDSSAT v1.0 -^98,104^
PEGASUS	Ecosystem	RUE, TE	W, T, H, N, P, K	Global	β	v1.1 -^106^, v1.0 -^105^
PEPIC	Site-based	RUE, TE	W, T, A, N, P, BD, AL	Site-specific and global	F HIpot (for maize)	EPIC v0810 -^60,108,125^
PRYSBI2	Ecosystem	LF, SC	W,T	Global yield	TH, TC, TS, LR	^114^

Notes: (NA where not applicable).

^a^Site-based: site-base crop model; Ecosystem: global ecosystem model.

^b^Elevated CO_2_ effects: LF: Leaf-level photosynthesis (via rubisco or quantum-efficiency and leaf-photosynthesis saturation; RUE: Radiation use efficiency; TE: Transpiration efficiency; SC: stomatal conductance.

^c^W: water stress; T: temperature stress; H: specific-heat stress; A: oxygen stress; N: nitrogen stress; P: phosphorus stress; K: potassium stress; BD: bulk density; AL: aluminum stress (based on pH and base saturation)

^d^F: fertilizer application rate; HIpot: Potential harvest index; LAImax: maximum LAI under unstressed conditions; HI: harvest index; αa: factor for scaling leaf-level photosynthesis to stand level; β: radiation-use efficiency factor; TH: Total Heat unit required for the maturity; TC: Technological coefficient; TS: Temperature sensitivity of photosynthesis; LR: ratio of leaf to above ground biomass.

**Table 3:** Crops simulated by the GGCMI phase 1 ensemble, abbreviations, and simulation priorities.

Crop	Species’ scientific names	Abbreviation	Simulation priority
Maize	Zea mays	mai	P1
Wheat	Triticum aestivum	whe	P1
Rice	Oryza spp.	ric	P1
Soybean	Glycine spp.	soy	P1
Barley	Hordeum vulgare	bar	P2
Millet	Various	mil	P2
Rapeseed	Brassica napus	rap	P2
Rye	Secale cereale	rye	P2
Sorghum	Sorghum spp.	sor	P2
Sugar beet	Beta vulgaris	sgb	P2
Sugar cane	Saccharum officinarum	sug	P2
Cotton	Gossypium hirsutum	cot	P2
Cassava	Manihot esculenta	cas	P2
Groundnut	Arachis hypogaea	nut	P2
Field pea	Pisum sativum	pea	P2
Sunflower	Helianthus annuus	sun	P2
Dry bean	Various	ben	P2
Potato	Solanum tuberosum	pot	P2
Managed grassland	Various	mgr	P2

### Modeling protocol

The overall modeling protocol is described by Elliott, *et al*.^37^ and we provide here only a summary of the main features. Modelers were asked to supply a minimum set of simulations, but could include additional simulations, addressing different directions of analysis. Online-only Table 1 lists all inputs used by the modeling groups. Modelers were asked to provide data for all simulated crops for all grid cells, even if crops are not currently cultivated in these areas. For these non-cultivated areas, input data were provided, but simulations could be skipped if no plausible assumptions on growing seasons could be made for that location^37^. According to the modeling protocol as described by Elliott, *et al*.^37^, modelers provided simulations for the default setup of their model (*default*), for harmonized growing seasons (i.e. prescribed grid-cell- and crop-specific sowing and maturity dates) and fertilizer inputs (*fullharm*) and for the same harmonized growing seasons but with unlimited nutrient supply (*harmnon*, also referred to as *harm-suffn* in some publications, e.g. Müller, *et al*.^38^). All simulations were conducted for purely rainfed (*noirr*) and for fully irrigated (*firr*) conditions as separate datasets so that crop yield could be aggregated with given crop- and irrigation masks to larger spatial entities in the post-processing^38,47,48^.

### Global Gridded Crop Models

The GGCMs contributing to the GGCMI phase 1 data archive differ in model structure, input requirements, and processes covered and thus have implemented the simulations in different ways. We here first describe each individual GGCM with central references and a short description of the model setup and conduction of simulations. In the following section, we provide tabular overviews of these narratives.

### CGMS-WOFOST

#### General description

CGMS-WOFOST (European Crop Growth Monitoring System with the WOrld FOod STudies crop simulation model)is a spatially distributed version of the WOFOST crop simulation model^49–51^. WOFOST is a mechanistic crop growth model that describes plant growth using light interception and CO_2_ assimilation as growth driving processes, and crop phenological development as a growth controlling process. The model can be applied using the following two modes: (1) a potential mode, in which crop growth is driven solely by temperature and solar radiation, and in which no growth limiting factors are taken into consideration; and (2) a water-limited mode, in which crop growth is limited by the availability of water. The difference in yields between the potential and water-limited modes can be interpreted as the effect of drought. Currently, no other yield-limiting factors (e.g. nutrients, pests, weeds, farm management) are taken into consideration.

WOFOST has been embedded in the European CGMS that was developed within the framework of the MARS (Monitoring Agricultural ResourceS project) project of the Joint Research Centre of the European Commission. CGMS allows the regional application of WOFOST by providing a database framework that handles model input (e.g. weather, soil and crop parameters), model output (crop indicators such as total biomass and leaf area index), aggregation to statistical regions and yield forecasting.

#### Model setup and protocol

The planting and harvest dates were taken from Elliott, *et al*.^37^ for the different crops. For each crop and grid cell a pre-run was executed in order to determine the temperature sum requirements (phenological heat units: PHU) from planting to harvest based on the 30-year AgMERRA (Agriculture Modern-Era Retrospective Analysis for Research and Applications) weather forcings^52^. This total PHU was then divided into a PHU from sowing to emergence, from emergence to anthesis and from anthesis to maturity based on the ratio of PHU values in the original WOFOST crop parameter files. As a result each grid cell receives its own variety definition in terms of temperature sum requirements for each of the 14 crops. The remaining model parameters were taken from the default WOFOST parameter files for each crop.

The cropping calendars provided by GGCMI are derived from regional sources (e.g. FAO which describe a static growing season for an entire region). However, many of those areas for which a growing season is defined do not have soil types that are suitable for crops. Therefore, grid cells for which a cropping calendar was defined but where soils are unsuitable were not simulated by CGMS-WOFOST. In practice, grid cells were excluded mainly in Northern Africa, Central Australia and Siberia.

Simulations with CGMS-WOFOST were carried out for the AgMERRA and WFDEI.GPCC weather forcings for all years within the range of the forcing set. The crop simulations for the irrigated scenario were executed with the WOFOST model running for the potential production scenario, assuming that crops were irrigated to the extent that no water stress occurs. The crop simulations for the rainfed scenario were executed with the model running in water-limited production scenario. For the latter scenario, large spin-up periods are not necessary as the model does not include simulation of carbon or nutrient pools. All simulations were started by starting the water balance calculation 90 days before the start of the crop sowing date with the water balance initialized at 50% of its water holding capacity (the range between wilting point and field capacity). This allows some time for the water balance to accumulate water as a result of rainfall. Finally, all 14 crops defined in GGCMI were simulated with CGMS-WOFOST for the two weather forcings mentioned above.

### CLM-Crop

#### General description

The CLM crop model was developed to improve the fully-coupled simulations of the Community Earth System Model (CESM1) and to help begin answering questions about changes in food, energy, and water resources in response to changes in climate, environmental conditions, and land use within the CESM modeling framework. CLM crop was initially incorporated into the CLM4CN model^53^ by replacing the unmanaged C3 crop plant functional type (using the C3 photosynthesis pathway) which represented all crops globally, with a small number of interactive managed crops over temperate northern hemisphere latitudes^54^. The CLM4CN crop model introduced the managed crop types of maize, soybean, and spring wheat (which represented more generally temperate cereals). The new crops reside on their own soil column, independent to the remaining natural vegetation that shares a single soil column. These crops were chosen based on the availability of their corresponding algorithms in the crop model AgroIBIS (agricultural version of the Integrated Biosphere Simulator)^55^. The main additions to CLM4CN involved the addition of the AgroIBIS crop phenology and allocation algorithms to those of the existing algorithms used for natural vegetation. In the CLM version 4.5 of the CLM crop model the standard CLM calculation of the parameter Vcmax25 (photosynthetic capacity at common temperature of 25 °C) was reintroduced to crops, along with new fertilizer management and nitrogen fixation by soybeans^56^. The fertilizer functionality adds a central U.S. annual crop specific amount of nitrogen directly to the soil mineral nitrogen pool for each crop. In the CLM post-4.5 version of the crop model used in the AgMIP GGCMI studies, extra tropical crops were added, to include sugarcane, rice, cotton, tropical maize, and tropical soybean using the CLM version 4.5 parameterizations with modified parameter values from Badger and Dirmeyer^57^. Specifically for sugarcane and tropical maize, functional form of temperate maize is used because all three are C4 plants (i.e. they use the C4 photosynthesis pathway). For tropical soybean the functional form of temperate soybean is used and for rice and cotton the spring wheat functional form is used.

#### Model setup and protocol

CLM Crop simulations were configured following the experimental design and initial conditions generated in the recent CLM Crop model investigations by Levis, *et al*.^58^. In those simulations the CLM post-4.5 model was spun up for 1050 years with repeated 1900–1920 meteorological forcing and an atmospheric CO_2_ mixing ratio of 299.7 ppm generated from a previous 20th Century CESM simulation contributed to the CMIP5 (Coupled Model Intercomparison Project Phase 5) effort^59^. Following the spin up period, a 20th Century simulation was performed from 1901–2005 using transient meteorological forcing and atmospheric CO_2_ generated from the same CESM simulation as used for the spin up. For AgMIP GGCMI all simulation configurations were started in 1978 with initial conditions provided for CLM crops from the 1978 state in the 20th Century simulation of Levis, *et al*.^58^. The CLM Crop AgMIP simulations were performed over the 1978–2010 period for rainfed and irrigated versions of cotton, maize, rice, sugar cane, soy and wheat crops. Temperate and tropical versions of maize and soy were represented separated by latitude, with tropical versions from 30°S–30°N and temperate versions outside of those latitudes. Meteorological forcing was generated for CLM on a 6-hour time step from the daily values provided by the AgMERRA and WFDEI datasets. The diurnal cycle for each of the reference height forcing variables (downwelling solar radiation, temperature, precipitation, pressure, specific humidity, and wind) were prescribed from the CLM CRU-NCEP (Climate Research Unit and National Centers for Environmental Prediction)6-hour forcing time series for the same period. For the harmonization simulations the annual nitrogen fertilizer applied was taken from spatially-varying crop-specific values provided by the AgMIP GGCMI protocol rather than the U.S. annual crop specific amount of the default model. Attempts to modify the planting dates and crop phenology were unsuccessful and so were not included in the study.

### EPIC-Boku

#### General description

EPIC-BOKU is a global grid-based modelling system based on the EPIC version 0810 model^60^. It contains routines for simulating crop growth and yield, hydrological, nutrient and carbon cycle, soil temperature and moisture, soil erosion and a wide range of crop management options. EPIC operates on a daily time step and can be used for long-term assessments. Potential plant growth is calculated based on intercepted solar radiation, conversion of CO_2_ to biomass and vapor pressure deficit. The potential growth is decreased by stresses imposed by temperature, nutrient deficit, salinity, aluminum toxicity, soil strength or aeration deficiency. Temperature stress occurs each day when average temperature exceeds the optimum temperature or falls below the base temperature, and water stress when soil water supply is insufficient to meet the potential plant evapotranspiration. Nutrient stress is calculated based on N (nitrogen) and P (phosphorus) deficit compared to optimal supply. Phenological development is based on daily heat unit accumulation that determines leaf area growth, canopy height, nutrient uptake, harvest index and, optionally, date of harvest. Crop yield is calculated from above-ground biomass and harvest index. EPIC incorporates equations that adjust radiation-use efficiency and evapotranspiration for elevated atmospheric CO_2_ concentration. The Penman-Monteith method was used to compute potential evapotranspiration.

#### Model setup and protocol

Global EPIC-BOKU was constructed within the “Global earth observation - benefit estimation: now, next and emerging” project of the Sixth Framework Programme of the European Commission to support integrated land-use modelling at global scale. It is run on a 5 arc-minutes grid by combining Geographic Information System layers on soils, relief, administrative units and a 0.5 arc-degrees (°) weather grid using the approach by Skalský, *et al*.^61^. Global crop simulations are performed on total cropland cover (GLC2000) stratified by homogenous response units at 5 to 30 arc-minutes grid resolution^61^, resulting in about 103,000 spatial modeling responses for cropland. The spatial modeling responses of crops and crop management variants are integrated in global economic land use models such as GLOBIOM^62,63^. The crops can be simulated for three management/input systems allowing to carry out the three GGCMI phase 1 configurations: 1) automatic nitrogen fertilization – N-fertilization rates based on crop specific N-stress levels (N-stress free days in 90% of the crop growing period). The upper limit of N application is 200 kg ha^−1^ a^−1^. 2) automatic nitrogen fertilization and irrigation – N and irrigation rates are based on crop specific stress levels (N and water stress free days in 90% of the crop growing period. N and irrigation upper limits of 200 kg ha^−1^ a^−1^ and 500 mm a^−1^). 3) subsistence farming – no N fertilizations and irrigation. All crops and management variants are simulated on total global crop land cover.

The GGCMI phase 1 protocol is applied to aggregate the spatial modeling responses of maize, rice, soybeans, and wheat to 0.5° × 0.5° grids. Sowing dates and the length of the growing season were obtained from Sacks, *et al*.^64^. Planting and harvesting dates are considered as the earliest possible dates. The planting and harvest operations were automatically postponed if the required PHU had not been accumulated on the given day. PHUs were estimated based on Princeton (*default*) and WATCH (*fullharm* and *harmnon*) historical weather data. Amount of fertilizer (N, P) as well as planting and harvesting dates were harmonized according to the GGCMI phase 1 data and protocol.

### EPIC-IIASA

#### General description

EPIC-IIASA is a global grid-based modelling system based on the EPIC version 0810 model^60^, described above for the EPIC-BOKU model. In contrast to EPIC-BOKU, EPIC-IIASA used the Hargreaves method to calculate potential evapotranspiration, static computing of field water capacity and wilting point, the Cesar Izaurralde denitrification method^65^ and no water erosion was included. A detailed description of other differences in parameterization of fundamental biophysical routines in EPIC-IIASA and EPIC-BOKU are provided by Folberth *et al*.^46^.

#### Model setup and protocol

Sowing dates and the length of the growing season were obtained from Sacks, *et al*.^64^ for the *default* setup, and from the data provided by GGCMI phase 1 for the harmonized simulations. Harvesting dates are considered as the earliest possible dates of harvest. The harvest operations were automatically postponed if the required PHU had not been accumulated on the given day. PHUs were estimated based on Princeton (*default*) and WATCH (*fullharm*, *harmnon*) historical weather data.

The regions of spring and winter wheat were identified based on observed data by Sacks, *et al*.^64^, if available. Otherwise, the same rules as in Liu, *et al*.^66^ were applied, assuming that spring wheat is grown between 30°S and 30°N and winter wheat in regions with greater latitudes. For maize, a low-yielding cultivar with a harvest index of 0.35 was used in sub-Saharan Africa, while a harvest index of 0.5 was used for other regions. Maize with optimum temperature of 22.5 °C and a base temperature of 6 °C is used for temperate and colder regions in Europe and Russia, while an optimum temperature of 25 °C and a base temperature of 8 °C was used elsewhere. For rice, the harvest index and biomass-energy ratio were regionally modified based on Xiong, *et al*.^67^. All other crop growth parameters were left at the default values, which are based on literature.

In the *default* setup, crop-specific annual N and P fertilizer application rates were obtained from Balkovič, *et al*.^68^ and Mueller, *et al*.^69^ for European and other countries, respectively. P fertilizer was applied as a fixed amount together with tillage, while N dosing was triggered automatically based on plant requirements until the annual N application rate was fulfilled^70^. Irrigation was estimated based on the MIRCA2000 database using the automatic irrigation trigger in EPIC to supply water when the water stress exceeded 10% in one given day, with a maximum annual amount of 2000 mm.

### EPIC-TAMU

#### General description

The EPIC version 1102 model is a further development of EPIC version 0810 described above for the EPIC-BOKU and EPIC-IIASA models, with the same fundamental routines for mechanistically simulating soil-plant-atmosphere dynamics. Additional model capabilities include improved soil water balance methods^71^, denitrification methods^72^, perennial crop growth routines^73^, and soil health impacts of biochar^74^.

#### Model setup and protocol

Data were computed for 40,500 pixels for which appropriate weather, soil and crop calendar data were available. A one-year spin up period was simulated in addition to the simulated years of each weather dataset. Planting occurs on the first day following the prescribed sowing date in which soil temperature is at least 2 °C above the 8 °C base temperature. Harvest occurs once the specified heat units are reached. Heat units to maturity were calibrated from the prescribed crop calendar data, and were limited to values between 900 and 3800 to ensure reasonable bounds^75,76^. Pixels with no prescribed harvest dates were provided a “fast-maturing” crop that would reach maturity at 900 heat units. Fast-maturing pixels were harvested one year following planting if maturity was not reached beforehand. For simulations with full irrigation, a high (0.99) threshold plant stress trigger was used, with any single application of water ranging from 25–100 mm. N, P and K (potassium) were applied on the sowing date based on the prescribed values for each site. For the *harmnon* runs, additional N was applied when a 0.99 threshold was triggered during the growing season. Additional N was added in increments of 25 kilograms per hectare. The Penman-Monteith method was used to compute evapotranspiration.

### GEPIC

#### General description

GEPIC is based on the field-scale model EPIC v0810, which calculates potential biomass increase for each day of a defined growing season based on leaf-area index (LAI) and solar radiation, and subsequently reduces the potential to an actual biomass increase using the maximum stress out of water, temperature, nutrients, salinity and aeration as a correction factor. Key parameters for crops are base temperature, maximum temperature, maximum leaf area index and development of LAI over time, as well as an energy-biomass conversion coefficient describing the efficiency of photosynthesis. Besides plant growth, EPIC estimates changes in soil properties and nutrient cycles based on plant and soil management.

#### Model setup and protocol

Planting dates were estimated similar to the approach of Waha, *et al*.^77^, but with a simplified classification. Grid cells with <5 °C in the coldest month are defined as temperature limited, all other grid cells as precipitation limited. In precipitation-limited grid cells, crops are planted on the first day of the month after which four consecutive months provide the highest precipitation throughout the year. In T limited grid cells, planting dates for summer crops are defined by cumulating PHU starting from the coldest month of the year until a crop-specific germination threshold is met. For winter crops, the same process is carried out backwards from the coldest month of the year.

PHU were estimated from reported current sowing and harvest dates according to Sacks, *et al*.^64^ for the *default* setup and from the datasets provided by GGCMI phase 1 for the harmonized runs based on long-term climate averages.

We found that it is necessary to simulate depletion of soil nutrients in low-input regions like sub-Saharan Africa in order to represent current reported yields satisfactorily, as soil are usually highly depleted under such conditions due to continuous cultivation without sufficient nutrient replenishment and decreasing fallow durations^78^. An appropriate time-scale was found to be about 30 years. In order to represent this current state of soil depletion in the crop model, each decade was simulated separately with a run-time of 40 years, out of which only the last 10 years are used as a simulation result.

The choice of spring wheat and winter wheat is based on temperature thresholds published by Stehfest, *et al*.^79^: Winter wheat is planted in grid cells with a minimum temperature in the coldest month of the year of >−10 °C and <5 °C based on decadal monthly means. Winter wheat and spring wheat sowing areas change dynamically throughout the simulations period in each decade.

For maize, a dataset of national human development index for the year 2000 (retrieved from http://geodata.grid.unep.ch) is used for distinguishing low- and high-input countries. Maize with a high potential harvest index (corresponding to current hybrids) of 0.55 is planted in countries with a Human Development Index larger 0.8 and maize with a lower harvest index of 0.35 (corresponding to local conventional varieties^80^) in all other countries. For rice, the harvest index and maximum leaf area index were modified based on current literature^81^. All other crop growth parameters were left at the default values, which are based on literature and field trials^76,80,82^.

### LPJ-GUESS

#### General description

The model is the crop-enabled version of LPJ-GUESS (Lund Potsdam Jena General Ecosystem Simulator), described in Lindeskog, *et al*.^83^. Its implementation bears similarities to LPJmL as described in Bondeau, *et al*.^84^, but differs in several important aspects, including not being calibrated to observed country-level yields, a new phenology scheme, and a dynamic calculation of the PHU required for a crop to achieve maturity. Sowing dates are calculated dynamically following Waha, *et al*.^77^. The PHU sum needed for full development of a crop in a particular grid cell is calculated using a 10-year running mean of heat unit sums accumulated from the sowing date to the end of a sampling period (ranging from 190 to 245 days) derived from default sowing and harvest limit dates^83^. There is no differentiation between varieties other than PHU, except for wheat for which either spring or winter sowing varieties are selected, based on prevailing climate. Crops are harvested upon full development. This dynamic variation of PHU to climate effectively assumes a perfect adaptation of crop cultivar to the prevailing climate. N limitation is not explicitly accounted for in this version of the model, which precedes Olin, *et al*.^85^.

#### Model setup and protocol

Outputs have been computed for 59,191 pixels covering the entire ice-free land surface. Spin-up was for 30 years using the first 30 years of the input-data timeseries. Spin-up only influences the initialization of the sowing date and dynamic PHU algorithms. A full spin-up of soil carbon pools, as required for standard LPJ-GUESS simulations^86^ was not required as they do not feedback on crop yields in this model version. Simulations ran uninterrupted for the whole timeseries. Simulations did not consider nitrogen limitation explicitly in this simulation set, so data for the *fullharm* setting are not available but only the *default* and *harmnon* settings.

### LPJmL

#### General description

Simulations with LPJmL (Lund Potsdam Jena managed land) have been using the latest version available at that time as described by^77,84,87^, with the expanded soil implementation as described by Schaphoff, *et al*.^88^. The model computes daily gross primary production and autotropic respiration as a function of intercepted radiation, air temperature and water stress in a mechanistic way and allocates assimilates to the different organs as a function of phenological stage and water stress. Nitrogen dynamics are not considered explicitly in this version, which precedes the von Bloh *et al*.^89^.

#### Model setup and protocol

Data have been computed for 67,420 pixels (CRU land mask) with LPJmL from 1951-2099 in a transient simulation run, using a 200-year spin-up to initialize soil water and to bring soil temperatures into equilibrium (natural vegetation and soil carbon pools are neglected here, so the model spin-up simulation could be short), recycling the first 30 years of that time series for the spin-up phase.

National cropping intensities of the *default* runs have been calibrated to FAO statistics (1996–2000) as described by Fader, *et al*.^87^ but with a linear LAI-FPAR model for maize^90^ and maximum intensity levels for maize at a maximum LAI of 5. The *harmnon* runs were conducted without calibrated intensity settings but using a maximum LAI of 5 everywhere for all crops except wheat and sugarcane (maximum LAI = 7). The minimum root-to-shoot ratios at maturity were set to 10% based on insights from the AgMIP wheat^91,92^ and maize^93^ pilot studies. Sowing dates were computed as described by Waha, *et al*.^77^, but were kept constant after 1951. The model decides internally whether to grow winter or spring wheat on wheat areas. It has a preference for winter wheat, but if winters are too long, it will grow spring varieties^84^.

Simulations with LPJmL did not consider nitrogen limitation explicitly in this simulation set, so data for the *fullharm* setting are not available but only the *default* and *harmnon* settings. For the *harmnon* simulations prescribed planting days were used directly as input. To compute (PHU requirements for the parameterization of observed maturity dates per crop and grid cell, the model was run once with the WATCH climate data, prescribing varieties that never mature and recording accumulated PHU on the prescribed maturity date. From this run, the average 1972–2001 PHU requirements were extracted and prescribed in the *harmnon* runs, so that maturity dates vary between years but on average are consistent with prescribed maturity dates. Variety traits other than those determining the growing season length were not varied in space or time.

### ORCHIDEE-crop

#### General description

Simulations with ORCHIDEE-crop (Organising Carbon and Hydrology In Dynamic Ecosystems crop model) have been conducted using an improved version from Wu, *et al*.^94^. The improvements include an allocation scheme resolving the source-sink regulation on biomass and yield, an irrigation scheme, and a fertilizer scheme. These updated developments are documented in Wang^95^.

#### Model setup and protocol

Simulations were performed over global land grids according to the land-sea mask of each climate input dataset. One-year spin-up was performed to balance the soil water budget. The time length of the spin-up was selected after testing the turnover time needed to balance the water budget of the 11-layer soil hydrology module in the model.

No calibration was made for GGCMI simulations. However, during development of ORCHIDEE-crop, the wheat and maize parameters were evaluated and calibrated against several agricultural eddy flux sites over Europe^94^. The rice phenology parameters were calibrated against phenological observation networks in China^96^.

For the *fullharm* scenario, all input data instructed in the protocol were used. For the *default* simulation, the nitrogen fertilizer map was derived from a combination dataset of FAO and the International Fertilizer Association^97^, which is static and crop-specific. For the *harmnon* scenario, an over-saturation rate of 500 kgN ha^−1^ was applied in order to eliminate nitrogen constraints.

### pAPSIM

#### General description

The pSIMS platform^98^ leverages high-performance computing resources at the University of Chicago and Argonne National Lab. It comprises an assortment of survey-based and geospatial data sources, and field-scale crop models, including those based in the *APSIM*^99^ (referred to as pAPSIM, the parallel version of the Agricultural Production Systems sIMulator), to simulate food, fiber and biomass production systems at high spatial resolution and continental or global extents.

#### Model setup and protocol

The *default* set-up was based on fixed planting dates for each grid cell from the Sacks *et al*. crop calendar^64^, with additional detail in the conterminous US provided by crop calendar data of the US Department of Agriculture^100^. All crops were first simulated using a range of cultivar phenology parameters and the cultivar which best reproduced the harvest dates from the Sacks *et al*. crop calendar^64^ was selected to be used in the *default* set-up. For maize, grid cells described in the SPAM2000 (spatial allocation model) dataset^101^ as “rainfed high input” or “irrigated” were assumed to use high-yielding hybrid cultivars, parameterized with 50% higher max grain number and 10% higher grain filling rate. Fertilizer levels in the *default* setting were the same as those used in the harmonized scenario (*fullharm*)^37^, with half applied at planting and half applied 40 days later. Wheat cultivar groups were selected based on mega-environments^102^ and then phenology parameters were calibrated as with other crops. Soybean cultivars were selected based on standard maturity groups and were then calibrated to reproduce Sacks *et al*.^64^ harvest dates^3^. In the *fullharm* and *harmnon* settings, growing periods of all crops were calibrated in the same manner to reproduce given GGCMI growing periods^37^.

The simulation period was reinitialized each year on January 1^st^ assuming a 50% full soil water profile in each location. Soil dynamics typically stabilized at expected levels before planting, though some caution must be taken for locations with planting very early in the calendar year (e.g. before the end of January). All other crop growth parameters were left at default values.

### pDSSAT

#### General description

The pSIMS platform^98^ leverages high-performance computing resources at the University of Chicago and Argonne National Lab. It comprises an assortment of survey-based and geospatial data sources, and field-scale crop models, including those based in the Decision Support System for Agrotechnology Transfer (DSSAT) framework (*CROPGRO*^103^ and *CERES* (Crop Environment Resource Synthesis^104^)) (referred to as pDSSAT, the parallel version of the Decision Support System for Agrotechnology Transfer), to simulate food, fiber and biomass production systems at high spatial resolution and continental or global extents.

#### Model setup and protocol

The model setup and protocol is identical to that of pAPSIM described above as both models are run by the same group in the pSIMS environment^98^.

### PEGASUS

#### General description

PEGASUS (Predicting Ecosystem Goods And Services Using Scenarios Model) combines a radiation use efficiency model to estimate daily photosynthesis and annual net primary production with a surface energy and soil water budget model. In addition, the model uses a dynamic allocation scheme to assign daily biomass production to the different organs of the crop. Thus, crop yield is eventually derived from the amount of carbon contained in the storage organs at harvesting date^105^. PEGASUS 1.1 simulates crop response to elevated CO_2_ and effects of extreme temperature events occurring at crop anthesis. A specific heat stress factor is calculated as a function of intensity and duration of extreme temperature events during crop anthesis according to crop specific temperature thresholds^106^. Farm management practices represented in PEGASUS include irrigation and fertilizer application, decision of planting dates and choice of crop cultivars^105^.

For the GGCMI phase 1 simulations, PEGASUS version 1.1 was used^106^.

#### Model setup and protocol

PEGASUS was calibrated to match average crop yields around the year 2000 of the Monfreda *et al*. dataset^107^, using a subset of the WATCH data (6 years from 1997 to 2002). Note that the calibration procedure in PEGASUS entails tuning only one global parameter, the light-use-efficiency coefficient (ε) as described in Deryng, *et al*.^105^.

For the *default* simulations, the calibrated version of PEGASUS from the ISIMIP fast-track^3^ was used, making use of PEGASUS’ internal algorithm to simulate planting date decision and choice of crop cultivars, as well as fertilizer data as referenced in Deryng, *et al*.^105^. This means that the *default* configuration allows for progressive adaption of planting dates and choice cultivars according to annual mean climate conditions.

For simulations of the *fullharm* and *harmnon* settings, a new calibrated version was used, using the same WATCH dataset as climate input and average crop yields around the year 2000 Monfreda *et al*. dataset^107^, but using: the harmonized crop calendar dataset and the harmonized fertilization application rates as specified by Elliott, *et al*.^37^. However, this calibrated version differs only for wheat, for which ε was set to 0.029 mol C m^−2^ s^−1^ APAR, instead of 0.027 mol C m^−2^ s^−1^ APAR. APAR (mol quanta m^−2^ s^−1^) represents the daily average absorbed photosynthetically active radiation. ε = 0.035 mol C m^−2^ s^−1^ APAR for maize and ε = 0.011 mol C m^−2^ s^−1^ APAR for soybean was used for both *default* and *fullharm* versions.

For this set of simulations, climate data were provided in one time-slice so that PEGASUS was run continuously over each time-period, including an initial 4-year spin-up. PEGASUS was run with downwelling longwave radiation input from the WFDEI dataset for AgMERRA and AgCFSR (Agriculture Climate Forecast System Reanalysis) simulations.

### PEPIC

#### General description

PEPIC (Python based EPIC model) is a grid-based EPIC model compiled under the Python environment^108^. The EPIC model was initially introduced by Williams, *et al*.^109^ to evaluate the impacts of soil erosion on soil productivity. EPIC can be used to simulate a large number of soil-water-climate-management processes, for example, weather, hydrology, erosion, pesticide, nutrient, plant growth, tillage, soil temperature, and environmental control^109^. EPIC simulates crop growth at a daily step based on the concept of energy-biomass conversion. Daily potential biomass increase is the product of intercepted solar radiation and a crop-specific biomass-energy ratio. Several crop growth stresses (water, nutrient, temperature, aeration, and salinity) are considered to reduce the potential biomass to actual biomass. The crop grain yield is estimated by the product of the harvest index and actual biomass accumulation^60^.

#### Model setup and protocol

In PEPIC, the whole study domain is firstly categorized into a number of subareas depending on the study purposes (e.g. administrative boundaries, climate regions, watersheds). Input data need to be specified for each grid cell with a spatial resolution of 30 arc-minutes. After all simulations are completed for all grids cells, PEPIC extracts the results and presents the spatial distribution of desired variables for a given time period. Irrigated and rainfed crop cultivations are simulated separately. To get combined outputs for each grid cell, values from irrigated and rainfed cultivation were aggregated using an area-weighted averaging method.

Potential heat units are calculated with a PHU calculator from the SWAT (Soil Water Assessment Tool) website (https://swat.tamu.edu/software/), with input of planting date, growing season length, and monthly minimum and maximum temperature. In the simulation, different PHUs have been computed for each weather forcing dataset. For *default* setup, crop calendar data (planting and harvesting dates) were derived from Sacks, *et al*.^64^, and N and P fertilizer from FertiSTAT (database for statistics on fertilizer use by crop)^110^ were used. For the harmonized setups (*fullharm* and *harmnon*), crop calendar, N, P and K fertilizer from GGCMI were used^37^.

For the simulation forced by each weather forcing dataset, 20 years were treated as model spin-up period. Automatic irrigation was used for irrigated cultivation with sufficient water supply (maximum value of 1000 mm). For *default* and *fullharm* scenarios, P was applied directly prior to planting and N was applied three times based on input data: first time before planting, second time one month after germination, third time two months after germination. One third of N inputs were applied for each application. For the *harmnon* scenario, N was applied automatically based on crop N requirement, with a stress trigger of 0.99 and sufficient N inputs. Similar to N fertilization under the *harmnon* scenario, P inputs were also determined by the model without limitation.

For cultivars of wheat and maize, PEPIC adopted the same approach as GEPIC (Section 2.2.6) to distribute the cultivar distribution globally. Rice and soy used the *default* parameters from the EPIC model.

### PRYSBI2

#### General description

The PRYSBI2 model (Process-based Regional-scale crop Yield Simulator with Bayesian Inference version 2.1) is a semi-process-based large-area crop model for major crops: maize, soybeans, wheat, and rice. Daily crop biomass growth and resulting crop yields are calculated for each global grid (1.125° in latitude and longitude). The daily biomass growth is calculated according to photosynthetic carbon assimilation based on the enzyme kinetics model (i.e. Farquhar model^111^). A sun/shade model^112^ is used for the calculation of intercepted solar radiation. The soil water balance is calculated by the SWAT model^113^. The crop development is calculated via PHU, as in the EPIC model. Daily temperature affects crop growth through mainly the changes in phenology, photosynthetic rate, and evapotranspiration rate. Daily precipitation affects crop growth through water stress calculated according to the SWAT model. Crop yield is calculated from above-ground biomass and a harvest index. The model (version 2.0) is described by Sakurai, *et al*.^114^.

We refer to this model as “semi-process-based” because the model parameters relevant to the past technological trend (i.e. it includes the past change of nutritional input, crop variety, and the degree of the irrigation etc.) were inversely estimated using historical crop yield data^115^ for each spatial grid using Markov Chain Monte Carlo methods. As such, the processes of fertilizer input and irrigation are not explicitly included put part of the inverse parametrization.

The version of the model is 2.1. From the version 2.0^114^, mainly following processes were changed.

1. The big leaf model was replaced by a sun/shade model^112^.

2. The calibrated technological factor no longer affects final biomass, but now affects daily biomass growth.

3. The estimated parameter set has been re-calibrated.

The PRYSBI2 model used here should not be confused with PRYSBI1 (an older version)^116^ which has a fundamentally different model structure.

The PRYSBI2 output was interpolated to the requested 0.5° resolution from its original 1.125° resolution at which simulations were conducted. This means that the output of a 0.5° grid was the same as the 1.125° grid in which the 0.5° grid was included. If a 0.5° grid straddled multiple 1.125° grids, the average value of these 1.125° grids was used. PRYSBI2 data do not distinguish irrigated and rainfed production as irrigation is subsumed in the technology factor as described above.

#### Model setup and protocol

The parameters relevant to the technological factor, including the temporal change rate of the technological factor and irrigation, were inversely estimated using historical crop yield data^117^ for each grid cell and crop using the DREAM (DiffeRential Evolution Adaptive Metropolis) algorithm^118^. The number of Markov Chain Monte Carlo steps was set to 50,000 for each grid cell. This large amount of calculation (about 3 × 10^9^ simulations in total) was executed on the super computer system of the Japan Agency for Marine-Earth Science and Technology (JAMSTEC).

In PRIBY2, the parameter values for the grid cells for which the reference data do not exist were extrapolated using the relationship between (1) the parameter values estimated at the grid cells in which the reference data exist and (2) environmental factors, such as elevation, harvested area, latitude, longitude, irrigated area^119^, planting day^64^, and the value of gross domestic product.

The dataset of Sacks, *et al*.^64^ was used for parametrizing the planting date in the *default* setup. The parameter set that has the maximum likelihood to reproduce observed yield dynamics^115^ for each grid and each crop was used for the *default* run. The simulation was set up to include one spin-up year before the first year of the simulation, using the weather data of the first simulation year. No other spin-up procedure was conducted, which was the same setting as in the calibration procedures (to reduce calculation time).

### GGCM configurations, calibration and evaluation

We distinguish two GGCM types: (i) site-based process models, and (ii) ecosystem models (Table 2). In addition to the models’ main characteristics (Table 2), Tables 4,5,6 provide overviews of agricultural practices and inputs used (Online-only Table 1), the most important biophysical processes implemented (Online-only Table 2), and calibration procedures (Table 4). Site-based models have typically been calibrated at field-scale level in previous model applications. Some of the site-based models were also calibrated at national scale, especially those EPIC models used to provide data to global or national economic analyses (EPIC-BOKU^62,120,121^, EPIC-IIASA^122^). Ecosystem models were either calibrated at national scale (LPJmL, PEGASUS) or not at all. An exception is PRYSBI2, which was extensively calibrated at grid-cell level. Generally, calibration of global-scale crop models is complicated by the lack of high-quality data and the absence of data on any aspect other than yield. Furthermore, calibration does not substantially improve model skill in global-scale applications, other than improving the reproduction of spatial patterns by imposing management-driven differences in yield levels in the calibration process^38^.

**Table 4:** Model calibration, evaluation, parameters, scale and methods.

Model	Model origin^a^	Calibration method	Parameters for calibration^b^	Output variable and dataset for calibration^c^	Spatial scale of calibration	Temporal scale of calibration	evaluation of simulated yields^d^
CGMS-WOFOST	Site-based	Default parameters from site-specific analysis (WOFOST 6.0)	NA	NA	Field scale	Mostly trials from 1980-2000	Müller, *et al*.^38^, Dobermann, *et al*.^222^, Bregaglio, *et al*.^223^, Boogaard, *et al*.^224^, Todorovic, *et al*.^225^, Eweys, *et al*.^226^, Setiyono, *et al*.^227^, Lecerf, *et al*.^228^, de Wit, *et al*.^229^
CLM-crop	Ecosystem	NA	NA	NA	NA	NA	Müller, *et al*.^38^, Drewniak, *et al*.^128^
EPIC-BOKU	Site-based	Site-specific (EPIC 0810)	NA	Yield (FE & FAO)	Field scale & National	Various	various, e.g. Müller, *et al*.^38^, Wang, *et al*.^230^, Wang, *et al*.^231^
EPIC-IIASA	Site-based	Site-specific (EPIC 0810) & Global^e^	F, HIpot (ric, mai)F (others)	Yield (FE & FAO)	National	Around 2000	various, e.g. Müller, *et al*.^38^, Wang, *et al*.^230^, Wang, *et al*.^231^
EPIC-TAMU	Site-based	Site-specific	HIpot (mai)	Yield (SPAM 2000 by You, *et al*.^134^)	Grid cell level	2000	Müller, *et al*.^38^, Izaurralde, *et al*.^65^
GEPIC	Site-based	Site-specific (EPIC 0810)^e^	NA	Yield (FE & FAO)	Field scale & National	Various	various, e.g. Müller, *et al*.^38^, Folberth, *et al*.^78^, Wang, *et al*.^230^, Wang, *et al*.^231^
LPJ-GUESS	Ecosystem	Uncalibrated	NA	NA	NA	NA	Müller, *et al*.^38^, Pugh, *et al*.^124^
LPJmL	Ecosystem	Global	LAImax HI alpha_a	Yield (FAO)	National	Average for 1998-2003	Müller, *et al*.^38^, Bondeau, *et al*.^84^
ORCHIDEE-crop	Ecosystem	Uncalibrated	NA	NA	NA	NA	Müller, *et al*.^38^, Wu, *et al*.^94^
pAPSIM	Site-based	Default parameters from site-specific analyses	NA	NA	field scale	NA	various, see http://www.apsim.info/, e.g. Müller, *et al*.^38^, Gaydon, *et al*.^232^
pDSSAT	Site-based	Site-specific (DSSAT)	NA	Yield (FE)	Field scale	Various	various, e.g. Müller, *et al*.^38^, Jones, *et al*.^104^
PEGASUS	Ecosystem	Global	Β	Yield (Monfreda, *et al*.^107^)	Grid cell level (0.5° lon × 0.5° lat resolution)	Average for 1997-2004	Müller, *et al*.^38^, Deryng, *et al*.^106^
PEPIC	Site-based	Default parameters from site-specific analyses of EPIC0810	NA	Yield (FAO yield statistics)	National	Average for 1998-2002	various, e.g.Müller, *et al*.^38^, Liu, *et al*.^108^, Wang, *et al*.^230^, Wang, *et al*.^231^
PRYSBI2	Ecosystem	Global	TH, TC, TS, LR	Yield (Iizumi, *et al*.^115^)	Grid cell level	1982-2006 (but the odd-numbered years were used as learning data for the estimation for the even years, and vice versa)	Müller, *et al*.^38^, Sakurai, *et al*.^114^

Notes: (NA where not applicable).

^a^Site-base crop model, ecosystem: global ecosystem model.

^b^F: fertilizer application rate; HIpot: Potential harvest index; LAImax: maximum LAI under unstressed conditions; HI: harvest index; αa: factor for scaling leaf-level photosynthesis to stand level; β: radiation-use efficiency factor; TH: Total Heat unit required for the maturity; TC: Technological coefficient; TS: Temperature sensitivity of photosynthesis; LR: ratio of leaf to above ground biomass.

^c^FE: field experiments; FAO: FAOSTAT national yield statistic;

^d^references to evaluation studies at global scale or to evaluation studies of the underlying site-based crop model at other scales. Often, only some examples are provided, given that model application studies also often provide some form of model evaluation.

^e^GEPIC & EPIC-IIASA: Default parameters coming with the field scale model EPIC version 0810 are mostly used. Potential HI has been adjusted for maize cultivars based on literature and the Human Development Index (GEPIC) or major world regions (EPIC-IIASA) and for rice based on literature. Fertilizer application rates have been modified for few countries that report very high yields and low fertilizer use, whereas most of these countries are known for their intensive use of manure.

**Table 5:** Weather datasets used to drive simulations in GGCMI phase 1.

Dataset	File name abbreviation	Reanalysis	Years	Resolution^a^	Bias correction target^b^	Notes
AgCFSR^52^	agcfsr	CFSR^233^	1980–2010	0.3 (0.5/0.25)	CRU-TS3.1^234^/GPCCv6^235^/UDel^236^/SRB^237,238^/TRMM^239^/PERSIANN^240^/CMORPH^241^	No longwave radiation provided, use rlds from WFDEI
AgMERRA^52^	agmerra	MERRA^242^	1980–2010	0.5° × 0.66 (0.5/0.25)	CRU-TS3.1^234^/GPCCv6^235^/UDel^236^/SRB^237,238^/TRMM^239^/PERSIANN^240^/CMORPH^241^	No longwave radiation provided, use rlds from WFDEI
CFSR reanalysis^233^	cfsr	CFSR^233^	1979–2012	0.3 (N/A)	N/A	
ERA-I reanalysis^243^	erai	ERA-I^243^	1979–2012	0.75 (N/A)	N/A	
GRASP^244^	grasp	JRA-25^245^ & ERA-40^246^	1961–2010	1.125 (1.125)	CRU-TS3.10/3.10.01/3.00^234^ CRU-CL1.0^247^, SRB^237,238^	No longwave radiation provided, use rlds from Princeton GF version 2
GSWP^c^	gswp	20CR^248^	1901–2010	0.5°	GPCC, CRU, SRB	Versions of bias-correction targets unclear. Addresses variability at high frequencies (less than monthly)
Princeton GF^249^	princeton	NCAR Reanalysis1^250,251^	1948–2008	2.8 (0.5)	CRU-TS2.1^252^/SRB^237,238^/TRMM^239^, GPCP^253^	
Princeton GF version 2^249^	pgfv2	NCAR Reanalysis1^250,251^	1901–2012	2.8 (0.5)	CRU-TS3.1^234^, SRB^237,238^, TRMM^239^, GPCP^253^	
WATCH^132^ (WFD)	watch	ERA-40^246^	1958–2001	2.5 (0.5)	GPCCv4^254^, CRU TS2.1^252^	Only using the precipitation version, which is bias-corrected against GPCCv4, even though there is also a version available for CRU
WFDEI.CRU^131^	wfdei.cru	ERA-I^243^	1979–2009	0.75 (0.5)	CRU TS3.1/3.101/3.21^234^	
WFDEI.GPCC^131^	wfdei.gpcc	ERA-I^243^	1979–2009	0.75 (0.5)	CRU TS3.1/3.101/3.21^234^, GPCCv5^255^/v6^235^	

We provide references for all datasets, where available, and an overview of the datasets used to generate these. For details on how the bias-correction was conducted we refer the reader to the corresponding publications.

^a^This denotes the resolution of the underlying reanalysis dataset (and in parentheses the typical resolution of the key target data, temp and precipitation, used in the bias correction). All datasets will be standardized to a 0.5° × 0.5° spatial resolution in the GGCMI archives.

^b^Abbreviations used: CMORPH: CPC MORPHing technique; CRU: Climate Research Unit; GPCC: Global Precipitation Climatology Centre; GPCP: Global Precipitation Climatology Project; PERSIANN: Precipitation Estimation from Remotely Sensed Information using Artificial Neural Networks; SRB: Solar Radiation Budget; TRMM: Tropical Rainfall Measuring Mission; UDel: University of Delaware;

^c^The GSWP dataset is not documented in an article yet, but briefly described at http://hydro.iis.u-tokyo.ac.jp/GSWP3/exp1.html#initial-conditions (accessed 2018/12/29).

**Table 6:** Filename conventions for standardized model outputs.

Filename tag []	Values
[model]	pdssat, epic-iiasa, lpjml, etc. (see Table 2)
[climate]	watch, wfdei.gpcc, wfdei.cru, grasp, agmerra, agcfsr, Princeton (see Table 5)
[clim.scenario]	Hist (included for consistency with the ISIMIP file naming convention)^256^
[sim.scenario]	default_firr, fullharm_noirr, etc. (see Section 2)
[variable]	yield, pirrww, plant-day, anth-day, etc. (see Table 7)
[crop]	mai, soy, whe, ric, mil, sor, etc. (see Table 3)
[timestep]	Annual (included for consistency with the ISIMIP file naming convention)^256^
[start-year]_[end-year]	1958_2001, 1980_2009, 1980_2010, etc. (see Table 5)

GGCMs have been evaluated in various forms: individually at field and global scale (see examples in Table 4, but note that this list is far from exhaustive) or in model intercomparison exercises also at field^91–93^ and global scale^38^. Aspects other than yield have not been evaluated in GGCMI, even though some models have been assessed also for other output variables^65,72,83,94,123–130^.

### Input data

All input data that have been supplied to modelers for the simulations has been described by Elliott, *et al*.^37^ and are available for download at http://www.rdcep.org/research-projects/ggcmi. In addition to the nine weather datasets listed by Elliott, *et al*.^37^, modelers also supplied simulations for an updated version of the Princeton data (PGFv2) that span 1901 to 2012 as well as the GSWP3 (http://hydro.iis.u-tokyo.ac.jp/GSWP3/) dataset, which has been supplied by ISIMIP phase2a (Online-only Table 1). The complete set of weather datasets to drive the crop models thus comprises eleven historical datasets that are based on retrospective datasets and nine of these have been bias-corrected against different observation-based products, including CRU and GPCC (Global Precipitation Climatology Center). This broad set of input data is meant to cover the uncertainty introduced from different reanalysis products and different bias-correction methods. An analysis of the role of different weather input data for GGCMs’ skill to reproduce historic yield variability is still pending.

All weather variables are bias-corrected individually, and against different data products. The 2-m temperature is typically bias-corrected against different versions of the CRU dataset, but precipitation can be bias-corrected against CRU, GPCC or other targets. The WFEDI bias-correction provides 2 sets, in which only the precipitation bias-correction differs^131^, denoted as WFDEI.CRU and WFDEI.GPCC respectively, no other subversions of weather forcing datasets are included or used here (Table 5). With this approach to bias-correct individual weather variables, the physical consistency between variables is not necessarily maintained. As such, it also seems acceptable to supplement weather variables from one dataset to another, if not supplied by the latter. This is the case for downwelling long-wave radiation, which is not used by all GGCMs but only by some (Table 4) and which is also not supplied by all datasets (Table 5). Additionally, not all weather variables have been bias-corrected in the different weather datasets. For some, bias-correction targets are non-existent (e.g. wind speed), for others, the authors of the bias-corrected datasets decided to not bias-correct all variables, such as 2-m temperatures in the WATCH dataset, which was only corrected for elevation after interpolation^132^. In contrast, in the WFDEI datasets 2-m temperatures were corrected to CRU temperature averages and diurnal ranges. All bias-correction was applied at the monthly level.

Soil data were not supplied to modelers, who were requested to use their own soil input data. Acknowledging the importance of soil information for crop yield simulations^45^, it was not possible to harmonize soil parameters within phase 1 across the different GGCMs, given the diversity in soil input requirements (number of layers; chemical, physical, biological and specific parameters or variables per layer).

## Data Records

### Data format

Data come in netCDF4 (network Common Data Form 4) files, with a naming convention as in Elliott, *et al*.^37^, using only lowercase letters in file names, but properly capitalized letters in subfolders. Each file contains only a single output variable. Files are named following the GGCMI convention^37^ (Table 6):

$${[model]}_{-}{[climate]}_{-}{[clim.scenario]}_{-}{[sim.scenario]}_{-}{[variable]}_{-}[crop]{}_{-}{[timestep]}_{-}{[start-year]}_{-}[end-year].{\bf{nc4}}$$

In the data archive, each model has its own subfolder (proper capitalization of model names), which includes a subfolder for each climate dataset simulated, which again contain subfolders for each simulated crop, using the long crop name rather than the abbreviation used in the file name (Table 3).

### Data availability

Data are available at https://zenodo.org/ (see Online-only Table 4). The GGCMs have provided different output sets, covering different climate datasets, crops (Online-only Table 3), and output variables (Table 7). The overall GGCMI phase 1 dataset at https://zenodo.org/ is structured by GGCM and crop, which have been published as 86 individual packages (Online-only Table 4). Given that models have submitted data for different crops, there are 13 individually published datasets for CGMS-WOFOST^135–147^, 6 for CLM-Crop^148–153^, 4 for EPIC-Boku^154–157^, 4 for EPIC-IIASA^158–161^, 2 for EPIC-TAMU^162,163^, 4 for GEPIC^164–167^, 15 for LPJ-GUESS^168–182^, 13 for LPJmL^183–195^, 4 for ORCHIDEE-crop^196–199^, 4 for pAPSIM^200–203^, 6 for pDSSAT^204–209^, 3 for PEGASUS^210–212^, 4 for PEPIC^213–216^, and 4 for PRYSBI2^217–220^. All data are published under the Creative Commons Attribution 4.0 International (CC BY 4.0) license.

**Table 7:** Output variables supplied by GGCMs for all simulations sets these have provided (Online-only Table 3).

Variable	Variable names	Unit (comments)	pDSSAT	EPIC-Boku	EPIC-IIASA	GEPIC	pAPSIM	PEGASUS	LPJ-GUESS	LPJmL	CGMS-WOFOST	CLM-Crop	EPIC-TAMU	ORCHIDEE-crop	PEPIC	PRYSBI2
Crop yields	yield_ < crop>	t ha^−1^ yr^−1^ (dry matter)	X	X	X	X	X	X	X	X	X	X	X	X	X	X
Applied irrigation water^a^	pirrww_ < crop>	mm yr^−1^ (firr scenarios only)	X	X	X	X	X	X	X	X		X		X	X	
Total Above ground biomass yield	biom_ < crop>	t ha^−1^ yr^−1^ (daily or weekly if possible)	X	X		X	X	X	X	X	X	X	X	X	X	X
Actual growing season evapotranspiration	aet_ < crop>	mm yr^−1^ (growing season only)	X	X	X	X	X	X	X	X	X	X	X	X	X	X
Actual planting date	plant-day_ < crop>	day of year	X		X	X	X		X	X	X	X		X		
Days from planting to anthesis	anth-day_ < crop>	days from planting	X				X		X		X					
Days from planting to maturity	maty-day_ < crop>	days from planting	X		X	X	X		X	X	X	X		X		X
Nitrogen appl. Rate	initr_ < crop>	kg ha^−1^ yr^−1^		X	X	X	X					X	X	X	X	
Nitrogen leached	leach_ < crop>	kg ha^−1^ yr^−1^				X	X					X			X	
Soil carbon emissions	sco2_ < crop>	kg C ha^−1^					X									
Nitrous oxide emissions	sn2o_ < crop>	kg N2O-N ha^−1^					X									
Accumulated precipitation, planting to harvest	gsprcp_ < crop>	mm yr^−1^ (growing season only)	X		X	X	X		X	X		X				
Growing season incoming solar	gsrsds_ < crop>	w m-2 yr^−1^ (growing season only)	X				X		X	X		X				
Sum of daily mean temps, planting to harvest	sumt_ < crop>	deg C-days yr^−1^ (growing season only)	X				X		X	X		X				

^a^applied irrigation water is Potential Irrigation Water Withdrawal (PIrrWW) with the harmonized assumption of no losses during conveyance and application. It is different from Potential Irrigation water Use (PIrrUse), as the latter only includes fluxes of irrigation water to the atmosphere, while the applied water also includes water that remains in the soil or runs off (runoff).

## Technical Validation

All data submitted to the GGCMI phase 1 were tested by a set of quality check scripts. Data were tested for compliance with data formats, checking units (Table 7), variable naming (Table 7), file naming (Table 6), and space and time dimensions. Formatting errors led to rejection from the data base. Statistics on data ranges, spatial coverage with valid data points were reported to modeling groups, so that they could check and decide if the simulation data needed fixing.

## Usage Notes

The GGCMI phase1 simulation dataset was conducted with the objective to have as much spatial coverage globally for all crops as possible. As such, crops are also simulated in many regions, where these crops are not currently grown or cannot be currently grown. Growing season data were supplied for an as large area as possible^37^, with the intention to harmonize across models but not necessarily to suggest that cropping is possible during these periods. As such, management, soil and/or weather data at any given site may differ from conditions assumed for the corresponding grid cell and results should mainly be analyzed for larger spatial entities rather than individual sites. Any aggregation or analysis of these data should consider this caveat and either mask currently cropped areas with crop- and irrigation-specific masks^133,134^ or handle and interpret these data with the necessary caution. Since aggregation masks can affect results^47^ these should be selected carefully to fit the intended purpose.

Almost all data analyses already conducted focused on crop yields for which models have been evaluated individually and jointly^38^. All other output variables have not been evaluated in this context. Generally, all data from the GGCMI phase 1 archive should be subjected to plausibility checks. Analyses that are sensitive to outliers should test for extreme values that are likely to exist in rare cases. It is also advisable to generally assess the range of simulated data when conducting analyses with these data, which can provide an indication of the embedded uncertainty.

Despite the semi-automated quality control scripts that tested spatial coverage and data ranges of values in submitted files, not all errors in the output files provided by the modelers could be identified and/or corrected. All issues that were identified after utilization of the data in other publications as well as the corrections applied are described here. As such, simulation outputs of LPJ-GUESS and LPJmL were initially reported with an erroneous grid definition, in which all grid cells were shifted. In the LPJ-GUESS results all pixels were shifted one grid cell eastward and northwards, in the LPJmL data, all pixels were shifted one grid cell northwards. These erroneous data were used in the analyses of Müller, *et al*.^38^, Porwollik, *et al*.^47^, but corrected versions were used for Frieler, *et al*.^39^, Schauberger, *et al*.^41^. The data from pAPSIM and pDSSAT do not cover the full land surface, as the simulations were conducted with an incomplete land mask, missing part of the eastern coastlines. These data are not available and could not be supplied at a later stage. The output variables on growing season weather conditions (*sumt*, *gsrsds* and *gsprec*, Table 7) were not sufficiently clearly defined in the protocol^37^ and have thus been reported in an inconsistent manner. Outputs of pAPSIM and pDSSAT report average daily values, the other models report total growing season sums. LPJ-GUESS and LPJmL results for *sumt* have not included negative temperatures (°C) but only reported values above the crops’ base temperatures, whereas the other models included all values. Users are advised to compile their own growing season climate indicators using the weather input data (Table 5) and data on sowing and maturity dates (Table 7). CGMS-WOFOST provided wrong file names and dimensions for WFDEI.GPCC, which run until 2012 instead of 2010 and contain 2 empty elements for the last 2 time steps.

### ISA-Tab metadata file

Download metadata file

## Data Availability

The data of the GGCMI phase 1 simulation set were produced by the individual modeling groups using different GGCMs. The source code of these models is subject to different distribution policies and needs to be requested from the individual groups. The source code of the central quality check as well as some general aggregation and data-processing scripts are available at https://github.com/RDCEP/ggcmi/tree/phase1.

## Online-only Tables

**Online-only Table 1:** Model inputs and agricultural management practices

Model	Spatial scale	Temporal scale^a^	Climate input variables^b^	Soil input data^c^	Spin-up^d^	Planting date decision^e^	Crop cultivars^f^	Irrigation rules^g,h^	Fertilizer application^i^	Crop residue^j^
CGMS-WOFOST	0.5° lon × 0.5° lat	D	Ta Tmn Tmx P Rad Vap WS	FAO 1:5M DSMW AWC HYD	H2O (1)	Fixed planting day	PHU fixed	NA	NA	NA
CLM-crop	1° lon × 1° lat	6-hourly	T,P,WS,Q,SW, Rad	IGBP Global Soil Data Task 2000	NA	S	PHU+V	MIRCA 2000^219^	N	To litter pool
EPIC-BOKU	5” lon × 5”lat (default); 0.5° lon × 0.5° lat (harmonized)	D	Tmn, Tmx, P, Rad, RH, WS	ISRIC-WISE, ROSETTA,AWC, ALBEDO^257^, HYD^258^	Soil OM, C, NH3, NO3, H2O, P(1)	S (fraction of PHU), fixed planting window	PHU - fixed	90/100/500/50/208^h^ maximum applied irrigation: 500 mm yr-1	automatic N input (max 200 kg ha-1 yr-1) PK (national stat. IFA) dynamic application	No, can be simulated
EPIC-IIASA	5” lon × 5”lat (default); 0.5° lon × 0.5° lat (harmonized)	D	Tmn, Tmx, P, Rad, RH, WS	ISRIC-WISE; ROSETTA; AWC; HYD^258^						
	Soil OM, C, NH3, NO3, H2O, P, CR (50)	F (fixed planting window)	PHU, 3 cult for mai, 2 cult for wheat fixed	90/100/2000/500/0^h^	NP (sub-national stat by ^69^P timing: rigid; N timing: automatic (based on N stress)	No				
EPIC-TAMU	0.5° lon × 0.5° lat	D	Tmn, Tmx, P, Rad, RH, WS	ISRIC-WISE	Soil OM, C, NH3, NO3, H2O, P, CR (10)	S, planting delayed until 2 deg above base temp	PHU, 2 cultivars for mai	99/100/9999/100/25^h^	NPK at planting	No
GEPIC	0.5° lon × 0.5° lat	D	Tmn, Tmx, P, Rad, RH, WS	ISRIC-WISE	Soil OM, C, NH3, NO3, H2O, P, CR (20)	F (fixed planting window)	PHU, 2 cultivars for mai - fixed	90/100/2000/ 1000/0^h^	NP (national stat. FertiSTAT), dynamic application of N, rigid application of P	Yes, Crop-specific
LPJ-GUESS	0.5° lon × 0.5° lat	D	Ta, P, Rad	HWSD, STC HYD^259^, THM^260^	H2O (30)	S^77^, fixed planting window for harmnon simulations	PHU+V (whe, sunfl, rapes); PHU (others) + clim. adap; BT	200/90/100/100^g^	NA	Yes, does not affect yield
LPJmL	0.5° lon × 0.5° lat	D	Ta, P, cld (or Rad)	HWSD, STC HYD^259^, THM^260^	H2O, Tsoil (200)	S^77^, fixed planting day after 1951	PHU+V (whe, sunfl, rapes); BT (mai); static (others) - fixed	300/90/100/varies^g^	NA	Yes, does not affect yield
ORCHIDEE-crop	0.5° lon × 0.5° lat	Half-hourly	Tmn, Tmax, P, Rad, RH, WS	NA	H2O (1)	F^64^	Fixed	200/90/100/varies^g^	N(IFA)	Yes, does not affect yield
pAPSIM	0.5° lon × 0.5° lat	D	Tmn, Tmx,P, Rad	HSWD	NA	F (S is also possible)	PHU and/or latitude, 2-3 for each cell	NA	GGCMI	NA
pDSSAT	0.5° lon × 0.5° lat	D	Tmn, Tmx, P, Rad	HWSD	Soil OM, C, NH3, NO3, H2O (1)	S^64^ fixed planting window	PHU and/or latitude, 2-3 for each cell - fixed	40/80/100/757 ric: 30/50/100/100^g^	GGCMI	Yes, does not affect yield
PEGASUS	0.5° lon × 0.5° lat	D	Ta, Tmn, Tmx, P, cld (or sun)	AWC (ISRIC-WISE)	H2O (4)	S^105^ clim. adapt	PHU + clim. adapt	40/90/100/100^g^	NPK (national stat. IFA), annual application	NA
PEPIC	0.5° lon × 0.5° lat	D	Tmn, Tmx,P, Rad, RH, WS	ISRIC-WISE	Soil OM, C, NH3, NO3, H2O, P, CR (20)	Fixed planting day	PHU, 2 cultivars for mai	90/100/1000/500/1^h^		
	NP (national stat. FertiSTAT), three times of N, rigid application of P	Yes								
PRYSBI2	1.125° lon × 1.125° lat	D	Tmn, Tmx, P, Rad, RH, WS	ISLSCP-II^261^	NA	F^64^	PHU - fixed	NA	NA	No

Notes: (NA where not applicable).

^a^D: daily time-step; M: monthly time-step; H: hourly time-step; WG: use monthly climate data interpolated to daily using a weather-generator.

^b^Ta: average temperature, Tmn: minimum temperature, Tmx: maximum temperature, cld: percentage of cloud cover, sun: fraction of sunshine hours; RH: relative humidity; WS: wind speed; Vap: vapour pressure, Rad: radiation.

^c^Source of soil property inputs (e.g., source of basic soil properties), plus method for manipulation to derive parameters required by the model); AWC: Available Water Capacity^262^; HYD: hydraulic soil parameters; THM: thermal parameters; HWSD: Harmonized world soil database^263^; STC: soil texture classification based on the USDA soil texture classification (http://ufdc.ufl.edu/IR00003107/00001); ISRIC-WISE^264^; ROSETTA^265^.

^d^Number of years for Spin up (x); OM: organic matter, C: carbon; NH3: ammonia; NO3: nitrate; H2O: soil water; P: phosphorus; CR: crop residues.

^e^S: Simulate planting dates according to climatic conditions; F: fixed planting dates; source of planting date data if applicable; PHU: potential heat unit; fixed planting window (i.e., does not allow for adaptation to climate change); clim. adapt: dynamic planting window (adaptation to climate change).

^f^PHU: Simulate crop PHU requirements according to estimated annual PHUs from daily temperature; Number of cultivars; PHU+V: PHU requirements and vernalization requirements computed based on past climate experience; BT base temperature computed based on past climate; fixed: static PHU requirement (no adaptation); clim. adapt: dynamic PHU requirement (adaptation to climate change).

^g^Irrigation rules: depth of soil moisture measured (cm) / critical lower soil moisture threshold to trigger irrigation event (%); / upper soil moisture threshold to stop irrigation (%); / irrigation application efficiency (%).

^h^Irrigation rules: EPIC-based models: water stress in crop to trigger automatic irrigation (%); / irrigation efficiency - runoff from irrigation water (%); / maximum of annual irrigation volume (mm); / maximum of single irrigation volume allowed (mm); / minimum of single irrigation volume allowed (mm).

^i^Fertilizer application, timing of application; NPK annual application of total NPK (nutrient-stress factor); source of fertilizer application data; timing: annual or dynamic; IFA: International Fertilizer Association.

^j^Remove residue or not (Yes/No).

**Online-only Table 2:** Biophysical process representation in GGCMs

Model	Leaf area development ^a^	Light interception ^b^	Light utilization ^c^	Yield formation ^d^	Stresses involved ^e^	Type of heat stress ^f^	Crop phenology ^g^	Type of water stress ^h^	Evapo-transpiration ^i^	Number of soil layers	Root distribution over depth ^j^	Soil CN model ^k^	CO_2_ effects ^l^
CGMS-WOFOST	DA	D	P-R	Prt	W T A	V	T DL V	S	PM	2	NON	NA	LF TE
CLM-crop	DA	D	P-R	Prt	W,N,H	V	T & DL	S	TF	10	EXP	C/N	LF, TE
EPIC-BOKU	PS	S	RUE	HIws Prt B	W T A N P BD AL	NA	T(HU) V O	E	PM	10	EXP W	C N B(1) P(6)	RUE TE
EPIC-IIASA	PS	S	RUE	HIws Prt B	W T A N P BD AL	NA	T(HU) V O	E	HAR	10	EXP W	C N B(1) P(6)	RUE TE
EPIC-TAMU	PS	S	RUE	HIws Prt B	W T A N P BD AL	NA	T(HU) V O	E	PM	3	EXP W	C N B(1) P(6)	RUE TE
GEPIC	PS	S	RUE	HIws Prt B	W T A N P BD AL	NA	T(HU) V O	E	HAR	5	EXP W	C N B(1) P(6)	RUE TE
LPJ-GUESS	DA	S	P-R	HIws	W T	NA	T V	E	PT	2	LIN	NA	LF, SC
LPJmL	PS	S	P-R	HI	W T	NA	T V	S	PT	5	EXP	NA	LF, SC
ORCHIDEE-crop	DA	S	P-R	Prt	WT N	VR	T(HU) DL O V	S	PT	11	EXP	NA	LF,SC
pAPSIM	DA	S	RUE,P-R(pasture only)	Gn, Prt, HIw (soy)	W N A H	V R F	T DL O V	E, S	TE	5	EXP	C,N,P,B(3)	RUE, TE, NE
pDSSAT	PS(soy=DA)	S /D	RUE/P-R	Gn	W T H A N	V R F	T V DL O	E	PT/PM	4	EXP	C N P(3)	RUE, LF, TE
PEGASUS	DA	S	RUE	Prt	W T H N P K	V F^m^	T(HU)	E	PT	3	LIN W	NA	RUE TE
PEPIC	PS	S	RUE	HIws Prt B	W T A N P BD AL	NA	T(HU) V O	E	PM	5	EXP W	C N B(1) P(6)	RUE TE
PRYSBI2	DA	S	P-R	HI	W T	V	HU	E	PM	2	EXP	NA	LF, SC

Notes: (NA where not applicable):

^a^DA: Dynamic simulation based on development and growth processes; PS: prescribed shape of LAI curve as function of phenology, modified by water stress & low productivity.

^b^S: Simple approach: D: Detailed approach.

^c^RUE: Simple (descriptive) radiation use efficiency approach; P-R: Detailed (explanatory) gross photosynthesis – respiration (for more details see Adam, et al.^266^).

^d^Yield formation depending on: HI: fixed harvest – index; B: total (above – ground) biomass; Gn: number of grains and grain growth rate; Prt: partitioning during reproductive stages; HIws: HI modified by water stress.

^e^W: water stress; T: temperature stress; H: specific-heat stress; A: oxygen stress; N: nitrogen stress; P: phosphorus stress; K: potassium stress; BD: bulk density; AL: aluminum stress (based on pH and base saturation).

^f^V: vegetative (source); R: reproductive organ (sink); F: number of grain (pod) set during the flowering period.

^g^Crop phenology is a function of: T: temperature; DL: photoperiod (day length); O: other water/nutrient stress effects considered; V: vernalization; HU: Heat unit index.

^h^E: ratio of supply to demand of water; S: soil available water in root zone.

^i^PM: Penman – Monteith; PT: Priestley –Taylor; HAR: Hargreaves; TE: transpiration efficiency; TF: Turbulent Flux^111^.

^j^LIN: linear; EXP: exponential; NON: no roots-just soil depth zone; W: actual roots depends on water availability in each soil layer.

^k^C model; N model; P(x): x number of organic matter pools; B(x): x number of microbial biomass pools.

^l^Elevated CO_2_ effects: LF: Leaf-level photosynthesis (via rubisco or quantum-efficiency and leaf-photosynthesis saturation; RUE: Radiation use efficiency; TE: Transpiration efficiency; SC: Stomatal conductance ; NE: Nitrogen use efficiency.

^m^see Deryng, *et al*.^106^.

**Online-only Table 3:** Availability of model output for the different weather input sets, harmonization settings and crops

		pDSSAT	EPIC-Boku	EPIC-IIASA	GEPIC	pAPSIM	PEGASUS	LPJ-GUESS	LPJmL	CGMS-WOFOST	CLM-Crop	EPIC-TAMU	ORCHIDEE-crop	PEPIC	PRYSBI2
**AgMERRA**	default	P1, mil, sor	P1	P1	P1	mai, whe, soy, sor	mai, whe, soy	P1, P2A	P1, P2B, mgr	P1, P2C	P1, sug, cot		P1	P1	P1
**AgMERRA**	harmnon	P1, mil, sor	P1	P1	P1	mai, whe, soy, sor	mai, whe, soy	P1, P2A	P1, P2B, mgr		P1, sug, cot	mai, whe	mai, whe, ric	P1	
**AgMERRA**	fullharm	P1, mil, sor	P1	P1	P1	mai, whe, soy, sor	mai, whe, soy				P1, sug, cot	mai, whe	P1	P1	
**WFDEI.GPCC**	default	P1, mil, sor	P1*	mai*, whe*, soy*	P1	mai, whe, soy, sor	mai*, whe*, soy*	P1, P2A	P1*, P2B*, mgr*	P1*, P2C*	P1*, sug*, cot*		P1	P1*	P1
**WFDEI.GPCC**	harmnon	P1, mil, sor	P1*	mai*, whe*, soy*	P1	mai, whe, soy, sor	mai*, whe*, soy*	P1, P2A	P1*, P2B*, mgr*		P1*, sug*, cot*	mai, whe	whe, ric	P1*	
**WFDEI.GPCC**	fullharm	P1, mil, sor	P1*	mai*, whe*, soy*	P1	mai, whe, soy, sor	mai*, whe*, soy*				P1*, sug*, cot*	mai, whe	P1	P1*	
**WATCH**	default	P1, mil, sor	P1	P1		mai, whe, soy, sor			P1, P2B, mgr						P1
**WATCH**	harmnon	P1, mil, sor	P1			mai, whe, soy, sor		P1	P1, P2B, mgr			mai, whe			
**WATCH**	fullharm	P1, mil, sor	P1	P1	P1	mai, whe, soy, sor	mai, whe, soy					mai, whe			
**AgCFSR**	default	P1, mil, sor	P1			mai, whe, soy, sor			P1, P2B, mgr					P1	P1
**AgCFSR**	harmnon	P1, mil, sor	P1	mai, whe, soy		mai, whe, soy, sor		P1	P1, P2B, mgr			mai, whe			
**AgCFSR**	fullharm	P1, mil, sor	P1	mai, whe, soy	P1	mai, whe, soy, sor	mai, whe, soy					mai, whe		P1	
**CFSR**	default	P1, mil, sor	P1			mai, whe, soy, sor			P1, P2B, mgr						
**CFSR**	harmnon	P1, mil, sor	P1			mai, whe, soy, sor		P1	P1, P2B, mgr			mai*, whe*			
**CFSR**	fullharm	P1, mil, sor	P1			mai, whe, soy, sor	mai, whe, soy					mai*, whe*			
**ERAI**	default	P1, mil, sor	P1			mai, whe, soy, sor			P1, P2B, mgr						
**ERAI**	harmnon	P1, mil, sor	P1			mai, whe, soy, sor		P1	P1, P2B, mgr			mai, whe			
**ERAI**	fullharm	P1, mil, sor	P1			mai, whe, soy, sor	mai, whe, soy					mai, whe			
**GRASP**	default	P1, mil, sor	P1			mai, whe, soy, sor			P1, P2B, mgr					P1	P1
**GRASP**	harmnon	P1, mil, sor	P1			mai, whe, soy, sor		P1	P1, P2B, mgr			mai, whe			
**GRASP**	fullharm	P1, mil, sor	P1	P1	P1	mai, whe, soy, sor						mai, whe		P1	
**Princeton**	default	P1, mil, sor	P1			mai, whe, soy, sor			P1, P2B, mgr				ric*		P1
**Princeton**	harmnon	P1, mil, sor	P1			mai, whe, soy, sor		P1	P1, P2B, mgr			mai, whe	mai*, soy*		
**Princeton**	fullharm	P1, mil, sor	P1			mai, whe, soy, sor	mai, whe, soy					mai, whe	mai*, ric*, soy*		
**WFDEI.CRU**	default	P1, mil, sor	P1			mai, whe, soy, sor			P1, P2B, mgr					P1	P1*
**WFDEI.CRU**	harmnon	P1, mil, sor	P1			mai, whe, soy, sor		P1	P1, P2B, mgr			mai*, whe*			
**WFDEI.CRU**	fullharm	P1, mil, sor	P1			mai, whe, soy, sor	mai, whe, soy					mai*, whe*		P1	
**PGFv2**	default		P1		P1*			P1, P2A	P1, P2B, mgr					P1	
**PGFv2**	harmnon		P1						P1, P2B, mgr						
**PGFv2**	fullharm		P1											P1	
**GSWP3**	default		P1					P1, P2A	P1, P2B, mgr				P1*	P1	
**GSWP3**	harmnon		P1												
**GSWP3**	fullharm		P1										mai*, whe*, soy*	P1	

P1 is the priority 1 crops (mai, whe, ric, soy), all other crops are or subsets are listed explicitly. Models that provide a long list of additional crops have these listed as GGCM-specific priority 2 crops (P2): P2A is bar, mil, rap, rye, sor, sgb, cas, nut, sun, ben, pot; P2B is mil, rap, sgb, sug, cas, nut, pea, sun; P2C is bar, mil, rap, rye, sor, cas, nut, sun, ben, pot. LPJmL also supplied data for managed grasslands (mgr), which is technically not a crop. Asterisks (*) indicate that the files deviate from the standard reporting time slice for that particular weather dataset. Abbreviations of crop names are as in Table 3.

**Online-only Table 4:** Table of data records

Dataset authors	Model	Crop	Citation	Dataset DOI
Hoek, S. & de Wit, A.	CGMS-WOFOST	Wheat	[133]	http://doi.org/10.5281/zenodo.1408517
Hoek, S. & de Wit, A.	CGMS-WOFOST	Sunflower	[134]	http://doi.org/10.5281/zenodo.1408519
Hoek, S. & de Wit, A.	CGMS-WOFOST	Soy	[135]	http://doi.org/10.5281/zenodo.1408521
Hoek, S. & de Wit, A.	CGMS-WOFOST	Sorghum	[136]	http://doi.org/10.5281/zenodo.1408523
Hoek, S. & de Wit, A.	CGMS-WOFOST	Rye	[137]	http://doi.org/10.5281/zenodo.1408525
Hoek, S. & de Wit, A.	CGMS-WOFOST	Rice	[138]	http://doi.org/10.5281/zenodo.1408529
Hoek, S. & de Wit, A.	CGMS-WOFOST	Rapeseed	[139]	http://doi.org/10.5281/zenodo.1408531
Hoek, S. & de Wit, A.	CGMS-WOFOST	Potato	[140]	http://doi.org/10.5281/zenodo.1408533
Hoek, S. & de Wit, A.	CGMS-WOFOST	Millet	[141]	http://doi.org/10.5281/zenodo.1408535
Hoek, S. & de Wit, A.	CGMS-WOFOST	Maize	[142]	http://doi.org/10.5281/zenodo.1408537
Hoek, S. & de Wit, A.	CGMS-WOFOST	Groundnut	[143]	http://doi.org/10.5281/zenodo.1408539
Hoek, S. & de Wit, A.	CGMS-WOFOST	Drybean	[144]	http://doi.org/10.5281/zenodo.1408541
Hoek, S. & de Wit, A.	CGMS-WOFOST	Barley	[145]	http://doi.org/10.5281/zenodo.1408543
Lawrence, P.	CLM-Crop	Wheat	[146]	http://doi.org/10.5281/zenodo.1409960
Lawrence, P.	CLM-Crop	Sugar Cane	[147]	http://doi.org/10.5281/zenodo.1409964
Lawrence, P.	CLM-Crop	Soy	[148]	http://doi.org/10.5281/zenodo.1409966
Lawrence, P.	CLM-Crop	Rice	[149]	http://doi.org/10.5281/zenodo.1409968
Lawrence, P.	CLM-Crop	Maize	[150]	http://doi.org/10.5281/zenodo.1409970
Lawrence, P.	CLM-Crop	cotton	[151]	http://doi.org/10.5281/zenodo.1409974
Schmid, E.	EPIC-Boku	Wheat	[152]	http://doi.org/10.5281/zenodo.1404761
Schmid, E.	EPIC-Boku	Soy	[153]	http://doi.org/10.5281/zenodo.1404763
Schmid, E.	EPIC-Boku	Rice	[154]	http://doi.org/10.5281/zenodo.1404765
Schmid, E.	EPIC-Boku	Maize	[155]	http://doi.org/10.5281/zenodo.1404767
Balkovič, J., Khabarov, N. & Skalský, R.	EPIC-IIASA	Wheat	[156]	http://doi.org/10.5281/zenodo.1403195
Balkovič, J., Khabarov, N. & Skalský, R.	EPIC-IIASA	Soy	[157]	http://doi.org/10.5281/zenodo.1403197
Balkovič, J., Khabarov, N. & Skalský, R.	EPIC-IIASA	Rice	[158]	http://doi.org/10.5281/zenodo.1403199
Balkovič, J., Khabarov, N. & Skalský, R.	EPIC-IIASA	Maize	[159]	http://doi.org/10.5281/zenodo.1403203
Reddy, A., Jones, C. & Izaurralde, R. C.	EPIC-TAMU	Wheat	[160]	http://doi.org/10.5281/zenodo.1409009
Reddy, A., Jones, C. & Izaurralde, R. C.	EPIC-TAMU	Maize	[161]	http://doi.org/10.5281/zenodo.1409013
Folberth, C.	GEPIC	Wheat	[162]	http://doi.org/10.5281/zenodo.1408571
Folberth, C.	GEPIC	Soy	[163]	http://doi.org/10.5281/zenodo.1408573
Folberth, C.	GEPIC	Rice	[164]	http://doi.org/10.5281/zenodo.1408575
Folberth, C.	GEPIC	Maize	[165]	http://doi.org/10.5281/zenodo.1408577
Pugh, T. A. M., Olin, S. & Arneth, A.	LPJ-GUESS	Wheat	[166]	http://doi.org/10.5281/zenodo.1408623
Pugh, T. A. M., Olin, S. & Arneth, A.	LPJ-GUESS	Sunflower	[167]	http://doi.org/10.5281/zenodo.1408625
Pugh, T. A. M., Olin, S. & Arneth, A.	LPJ-GUESS	Sugar Beet	[168]	http://doi.org/10.5281/zenodo.1408633
Pugh, T. A. M., Olin, S. & Arneth, A.	LPJ-GUESS	Soy	[169]	http://doi.org/10.5281/zenodo.1408629
Pugh, T. A. M., Olin, S. & Arneth, A.	LPJ-GUESS	Sorghum	[170]	http://doi.org/10.5281/zenodo.1408635
Pugh, T. A. M., Olin, S. & Arneth, A.	LPJ-GUESS	Rye	[171]	http://doi.org/10.5281/zenodo.1408637
Pugh, T. A. M., Olin, S. & Arneth, A.	LPJ-GUESS	Rice	[172]	http://doi.org/10.5281/zenodo.1408639
Pugh, T. A. M., Olin, S. & Arneth, A.	LPJ-GUESS	Rapeseed	[173]	http://doi.org/10.5281/zenodo.1408641
Pugh, T. A. M., Olin, S. & Arneth, A.	LPJ-GUESS	Potato	[174]	http://doi.org/10.5281/zenodo.1408643
Pugh, T. A. M., Olin, S. & Arneth, A.	LPJ-GUESS	Millet	[175]	http://doi.org/10.5281/zenodo.1408645
Pugh, T. A. M., Olin, S. & Arneth, A.	LPJ-GUESS	Maize	[176]	http://doi.org/10.5281/zenodo.1408647
Pugh, T. A. M., Olin, S. & Arneth, A.	LPJ-GUESS	Groundnut	[177]	http://doi.org/10.5281/zenodo.1408649
Pugh, T. A. M., Olin, S. & Arneth, A.	LPJ-GUESS	Drybean	[178]	http://doi.org/10.5281/zenodo.1408651
Pugh, T. A. M., Olin, S. & Arneth, A.	LPJ-GUESS	Cassava	[179]	http://doi.org/10.5281/zenodo.1408653
Pugh, T. A. M., Olin, S. & Arneth, A.	LPJ-GUESS	Barley	[180]	http://doi.org/10.5281/zenodo.1408655
Müller, C.	LPJmL	Wheat	[181]	http://doi.org/10.5281/zenodo.1403013
Müller, C.	LPJmL	Rapeseed	[182]	http://doi.org/10.5281/zenodo.1403064
Müller, C.	LPJmL	Millet	[183]	http://doi.org/10.5281/zenodo.1403066
Müller, C.	LPJmL	Managed Grass	[184]	http://doi.org/10.5281/zenodo.1403068
Müller, C.	LPJmL	Maize	[185]	http://doi.org/10.5281/zenodo.1403073
Müller, C.	LPJmL	Groundnut	[186]	http://doi.org/10.5281/zenodo.1403078
Müller, C.	LPJmL	Cassava	[187]	http://doi.org/10.5281/zenodo.1403085
Müller, C.	LPJmL	Field Pea	[188]	http://doi.org/10.5281/zenodo.1403083
Müller, C.	LPJmL	Sugar Cane	[189]	http://doi.org/10.5281/zenodo.1403050
Müller, C.	LPJmL	Sugar Beet	[190]	http://doi.org/10.5281/zenodo.1403052
Müller, C.	LPJmL	Soy	[191]	http://doi.org/10.5281/zenodo.1403054
Müller, C.	LPJmL	Rice	[192]	http://doi.org/10.5281/zenodo.1403060
Müller, C.	LPJmL	Sunflower	[193]	http://doi.org/10.5281/zenodo.1403048
Wang, X. & Ciais, P.	ORCHIDEE-crop	Wheat	[194]	http://doi.org/10.5281/zenodo.1408191
Wang, X. & Ciais, P.	ORCHIDEE-crop	Soy	[195]	http://doi.org/10.5281/zenodo.1408193
Wang, X. & Ciais, P.	ORCHIDEE-crop	Rice	[196]	http://doi.org/10.5281/zenodo.1408195
Wang, X. & Ciais, P.	ORCHIDEE-crop	Maize	[197]	http://doi.org/10.5281/zenodo.1408199
Elliott, J.	pAPSIM	Wheat	[198]	http://doi.org/10.5281/zenodo.1403183
Elliott, J.	pAPSIM	Soy	[199]	http://doi.org/10.5281/zenodo.1403185
Elliott, J.	pAPSIM	Sorghum	[200]	http://doi.org/10.5281/zenodo.1403187
Elliott, J.	pAPSIM	Maize	[201]	http://doi.org/10.5281/zenodo.1403189
Elliott, J.	pDSSAT	Wheat	[202]	http://doi.org/10.5281/zenodo.1403171
Elliott, J.	pDSSAT	Soy	[203]	http://doi.org/10.5281/zenodo.1403173
Elliott, J.	pDSSAT	Sorghum	[204]	http://doi.org/10.5281/zenodo.1403175
Elliott, J.	pDSSAT	Rice	[205]	http://doi.org/10.5281/zenodo.1403177
Elliott, J.	pDSSAT	Millet	[206]	http://doi.org/10.5281/zenodo.1403179
Elliott, J.	pDSSAT	Maize	[207]	http://doi.org/10.5281/zenodo.1403181
Deryng, D.	PEGASUS	Wheat	[208]	http://doi.org/10.5281/zenodo.1409546
Deryng, D.	PEGASUS	Soy	[209]	http://doi.org/10.5281/zenodo.1409548
Deryng, D.	PEGASUS	Maize	[210]	http://doi.org/10.5281/zenodo.1409550
Liu, W. & Yang, H.	PEPIC	Wheat	[211]	http://doi.org/10.5281/zenodo.1403205
Liu, W. & Yang, H.	PEPIC	Soy	[212]	http://doi.org/10.5281/zenodo.1403207
Liu, W. & Yang, H.	PEPIC	rice	[213]	http://doi.org/10.5281/zenodo.1403209
Liu, W. & Yang, H.	PEPIC	Maize	[214]	http://doi.org/10.5281/zenodo.1403211
Sakurai, G.	PRYSBI2	Wheat	[215]	http://doi.org/10.5281/zenodo.1404828
Sakurai, G.	PRYSBI2	Soy	[216]	http://doi.org/10.5281/zenodo.1404832
Sakurai, G.	PRYSBI2	rice	[217]	http://doi.org/10.5281/zenodo.1404838
Sakurai, G.	PRYSBI2	Maize	[218]	http://doi.org/10.5281/zenodo.1404836
